# Circulating serotonin is associated with calcific aortic valve stenosis: a two-phase metabolomics study

**DOI:** 10.3389/fphar.2026.1934998

**Published:** 2026-09-23

**Authors:** Wei Gao, Chenghu Guo, Nizal Sarrafzadegan, Haowei Zhang, Zeyuan Mei, Jiaqi Li, Lin Chen, Junkang Cheng, Jocelyn Gao, Chaoyu Liu, Yue You, Zhiyi Wang, Mei Dong, Mei Ni, Xiaolin Liu, Xiaoling Liu, Guipeng An

**Affiliations:** 1 State Key Laboratory for Innovation and Transformation of Luobing Theory, Key Laboratory of Cardiovascular Remodeling and Function Research of MOE, NHC, CAMS and Shandong Province, Department of Cardiology, Qilu Hospital of Shandong University, Jinan, China; 2 Isfahan Cardiovascular Research Center, Cardiovascular Research Institute, Isfahan University of Medical Sciences, Isfahan, Iran; 3 The Sidney Kimmel Medical College at Thomas Jefferson University, Philadelphia, PA, United States

**Keywords:** biomarker candidate, calcific aortic valve stenosis, diagnostic discrimination, metabolomics, prospective evaluation, serotonin

## Abstract

**Background:**

Calcific aortic valve stenosis (CAVS) is the most prevalent valvular heart disease in the elderly, yet no pharmacological therapy can halt its progression, partly due to incomplete understanding of disease-associated metabolic alterations and the lack of circulating biomarkers for disease detection.

**Objective:**

We aimed to characterize metabolic alterations associated with CAVS and evaluate a candidate circulating marker for CAVS detection.

**Methods:**

In the discovery cohort, patients with severe CAVS and matched controls without aortic valve disease were enrolled. Untargeted serum metabolomics was performed to characterize metabolic profiles. Differential metabolites, pathway enrichment analysis, and machine-learning-based feature prioritization were conducted. Based on pathway-level metabolic annotation, biological evidence, and feasibility of targeted measurement, serotonin was selected as a biologically prioritized, hypothesis-generating candidate and evaluated by ELISA in an independent targeted assessment cohort including controls, non-severe CAVS, and severe CAVS. Associations between serum serotonin levels and CAVS were assessed using multivariable logistic regression, smooth curve analysis, subgroup analyses, and ROC analysis.

**Results:**

The discovery cohort included 58 participants [29 with severe CAVS (69.7 ± 7.5 years; 44.8% male) and 29 matched controls (66.8 ± 5.1 years; 58.6% male)]. Untargeted metabolomics identified 70 differential metabolites. KEGG pathway enrichment analysis revealed serotonin-related metabolic annotations, while Random Forest analysis prioritized indoleacrylic acid as a discriminatory metabolite feature. Serotonin was not selected based on individual metabolite ranking but was evaluated based on pathway-level evidence, biological plausibility, and feasibility of targeted measurement. In the independent targeted assessment cohort of 303 individuals (101 controls, 101 non-severe CAVS, and 101 severe CAVS), serum serotonin levels were significantly lower in both non-severe and severe CAVS groups compared with controls, but did not differ between disease severity groups. Multivariable logistic regression adjusted for confounders confirmed its independent inverse association with CAVS. Incorporation of serum serotonin into the clinical model improved discrimination (AUC increased from 0.752 to 0.865), whereas serotonin alone showed moderate diagnostic performance.

**Conclusion:**

Reduced circulating serotonin is associated with the presence of CAVS and may represent a biologically plausible candidate adjunct biomarker for disease detection. Further multicenter prospective and mechanistic studies are required to determine its clinical utility and biological significance.

## 1 Introduction

Calcific aortic valve stenosis (CAVS) is a prevalent and increasingly important form of valvular heart disease in aging populations. The disease evolves from valve sclerosis to progressive leaflet calcification, restricted valve opening, pressure overload, myocardial remodeling, heart failure, and death if severe symptomatic disease is not treated (Dweck et al., 2012; Chen et al., 2024). Current management relies primarily on echocardiographic assessment of valve anatomy and hemodynamics, and valve replacement remains the definitive therapy for advanced disease (Thanassoulis et al., 2013; Tomova et al., 2020). Despite these advances, no pharmacological therapy has been proven to halt or reverse CAVS progression, underscoring the need for circulating biomarker candidates that can complement imaging and support biological stratification (Otto et al., 2021; Vahanian et al., 2022; Chen et al., 2024; Shelbaya et al., 2024).

CAVS is now understood as an active fibrocalcific disorder rather than a passive degenerative process. Endothelial injury, lipid infiltration, oxidative stress, chronic inflammation, extracellular vesicle signaling, osteogenic differentiation of valvular interstitial cells, and calcium-phosphate deposition interact across the disease course (Greenberg et al., 2022; Blaser et al., 2023; 2025; Small et al., 2024; Desai and Braunwald, 2025). Genetic and lipid-related evidence implicates low-density lipoprotein cholesterol (LDL-C), lipoprotein(a) [Lp(a)], oxidized phospholipids, and inflammatory signaling in valve calcification and stenosis progression (Tomova et al., 2020; Thanassoulis et al., 2013; Zheng et al., 2019; Smith et al., 2014). These mechanisms provide a biological rationale for studying circulating metabolic signatures that may reflect systemic and valve-related remodeling.

Metabolomics and broader omics approaches offer a data-driven strategy for identifying biochemical pathways and biomarker candidates in CAVS. Prior work has shown that metabolites related to nitric oxide metabolism, lipid homeostasis, bile acid signaling, and inflammatory pathways can distinguish patients with severe aortic stenosis from controls (Blaser et al., 2025; Lupieri, 2025; van Driel et al., 2021; Zhang et al., 2025). Recent proteomic and multiomic studies further support the concept that circulating and tissue-derived molecular profiles can capture disease biology beyond conventional clinical variables (Blaser et al., 2023; Shelbaya et al., 2024). However, most candidate markers remain exploratory, and their associations with disease presence, independence from clinical covariates, and reproducibility across disease stages require further evaluation.

Serotonin is biologically plausible in this context because it is synthesized largely in the gut, stored predominantly in platelets, and can influence cardiovascular and valvular biology through serotonergic signaling pathways (Mekontso-Dessap et al., 2006; Bouchareb et al., 2019; Fong et al., 2021; Holeček, 2026). Excess serotonergic signaling has been linked to valvulopathy and 5-HT2B receptor-mediated valvular interstitial cell mineralization, whereas alterations in circulating serotonin levels may reflect changes in platelet serotonin handling, local serotonergic signaling, or broader tryptophan-related metabolic remodeling, although the underlying mechanisms remain unclear (Bouchareb et al., 2019; Fong et al., 2021; Holeček, 2026). In this study, we applied untargeted metabolomics to characterize metabolic alterations associated with CAVS and subsequently evaluated circulating serotonin as a biologically prioritized candidate based on pathway-level metabolic annotation, prior biological evidence, and feasibility of targeted measurement. We further investigated whether serum serotonin levels were associated with CAVS presence and whether it provided additional discriminatory information beyond conventional clinical variables.

## 2 Methods

### 2.1 Study design and participants

This study consisted of a discovery cohort (untargeted metabolomic screening) and an independent targeted assessment cohort. Participants were enrolled at Qilu Hospital of Shandong University. In the discovery cohort, patients with severe CAVS and age- and sex-matched controls without aortic valve disease were initially recruited based on echocardiographic findings between February 2024 and April 2024. In the independent targeted assessment cohort, consecutive patients with CAVS were prospectively enrolled from August 2024 to June 2025 and classified into two groups: severe CAVS and mild-to-moderate (non-severe) CAVS. Age- and sex-matched individuals without echocardiographic evidence of aortic valve disease were consecutively screened and enrolled as controls during the same recruitment period from the same hospital. Controls were screened using predefined inclusion and exclusion criteria and were not selected through convenience sampling. The discovery cohort was used for untargeted metabolomic profiling, whereas the independent cohort was used for targeted evaluation of serum serotonin levels and assessment of its association with CAVS presence.

Inclusion criteria for non-severe CAVS were as follows: age 30–90 years; echocardiographic diagnosis of mild or moderate CAVS defined by aortic valve area (AVA) ≥ 1 cm^2^ and ≤3 cm^2^, or aortic valve maximal velocity (AV Vmax) ≥ 2.5 m/s and <4.0 m/s, or mean aortic valve gradient (mean AVG) > 0 mmHg and <40 mmHg (Otto et al., 2024). Inclusion criteria for severe CAVS were: age 30–90 years; AVA <1 cm^2^, or AV Vmax ≥4.0 m/s, or mean AVG ≥40 mmHg (Otto et al., 2024). Controls met the following: age 30–90 years; normal aortic valve structure and function confirmed by echocardiography (AVA >3 cm^2^ and AV Vmax <2.5 m/s). Exclusion criteria for all participants were: (1) severe hepatic dysfunction [alanine aminotransferase (ALT) > 10 times the upper limit of normal] or severe renal dysfunction [estimated glomerular filtration rate (eGFR) < 30 mL/min/1.73 m^2^]; (2) rheumatic heart disease; (3) infective endocarditis; (4) history of valve replacement or repair; (5) connective tissue disease; (6) history of malignancy.

The study adhered to the Declaration of Helsinki and was approved by the Research Ethics Committee of Qilu Hospital of Shandong University (Approval No.: KYLL-2025 (ZM)-906). All participants provided written informed consent.

### 2.2 Clinical data collection

The following clinical data were collected for each participant: demographic information (age, sex), blood pressure, BMI, smoking status, hypertension history, diabetes mellitus history, lipid disorder history, medication history (including β-blockers, calcium channel blockers, diuretics, hypoglycemic agents, lipid-lowering drugs, and antiplatelet agents), physical examination, echocardiographic parameters (peak transvalvular gradient, mean transvalvular gradient, peak aortic jet velocity, aortic valve area), blood routine examination (white blood cell, red blood cell, hemoglobin, platelet, neutrophil), and key blood biochemical parameters (alanine aminotransferase, aspartate aminotransferase, total cholesterol, high-density lipoprotein cholesterol, low-density lipoprotein cholesterol, triglycerides, Lp(a), homocysteine, free fatty acids, phospholipase A2, fasting blood glucose, creatinine, uric acid, eGFR). The estimated glomerular filtration rate (eGFR) was calculated using the four-variable Modification of Diet in Renal Disease (MDRD) equation: eGFR = 175 × (Scr)^−1.154^ × (Age)^−0.203^ × (0.742 if female), where eGFR is expressed as mL/min/1.73 m^2^, Scr represents serum creatinine concentration in mg/dL, and age is expressed in years. The race-specific coefficient was not applied because all participants were Asian.

### 2.3 Blood sample collection

Fasting venous blood samples (5 mL) were collected from each participant into serum separator tubes containing clot activator and gel. Concurrent complete blood counts, including platelet count (PLT), were obtained for all participants. Samples were allowed to clot for exactly 30 min at room temperature, then centrifuged at 3000 rpm for 15 min at 4 °C. The supernatant serum was harvested, aliquoted, and stored at −80 °C. All serum samples were visually inspected for hemolysis; hemolyzed samples were excluded. Aliquots underwent no more than one freeze–thaw cycle before measurement.

### 2.4 Untargeted metabolomics testing

Collected serum samples were thawed on ice. For each sample, 100 μL of serum was mixed with 400 μL of precooled methanol and incubated at −20 °C for 30 min. After centrifugation at 20,000 × g for 15 min, the supernatant was dried under vacuum and reconstituted in 100 μL of 80% methanol. A pooled quality control (QC) sample was prepared by combining 10 μL from each extraction mixture. All samples were stored at −80 °C until LC–MS analysis.

Chromatographic separation was performed on an ultraperformance liquid chromatography (UPLC) system (SCIEX, UK), and metabolite detection was carried out using a TripleTOF 5600+ high-resolution tandem mass spectrometer (SCIEX, UK) operated in both positive and negative ion modes. Mass accuracy was calibrated every 20 samples. Samples were analyzed in random order, with QC samples injected every eight runs to monitor data quality. LC–MS and untargeted metabolomics analyses were conducted at LC-Bio Technology Co., Ltd (Hangzhou, China).

Raw data were converted to mzXML format and processed using the XCMS, CAMERA, and metaX packages in R. Metabolites were annotated by matching m/z values against the KEGG and HMDB databases, with further validation using an in-house fragment spectrum library. Peak intensity data were preprocessed with metaX. Principal component analysis (PCA) was performed to assess overall data distribution and identify potential outliers and batch-related variation. A QC-based LOESS signal correction was applied to correct intensity drift over time. Metabolic features with a relative standard deviation (RSD) > 30% across QC samples were excluded.

### 2.5 Metabolomics and machine-learning analysis

Differences in metabolite levels between groups were initially assessed using Student’s t-test after appropriate data preprocessing. Untargeted metabolomic data were log-transformed and unit variance scaled before multivariate analysis. Partial least squares discriminant analysis (PLS-DA) was performed to evaluate global metabolic differences between CAVS patients and controls. Model performance was assessed using *R* ^2^ and Q^2^ statistics, and model robustness was evaluated using 200 permutation tests. Permutation-derived intercepts were used to assess the potential risk of overfitting, and permutation P values were calculated to determine whether the observed classification performance exceeded that expected by chance.

Differential metabolites in the discovery phase were identified based on a combination of statistical significance and biological relevance. The initial screening criteria included absolute fold change (|FC|) > 1.2, P < 0.05, and variable importance in projection (VIP) > 1. To evaluate statistical robustness in this high-dimensional dataset, Benjamini–Hochberg false discovery rate (FDR) correction was applied to all metabolic features included in the differential analysis. Because the discovery cohort was exploratory and relatively small (n = 58), FDR-adjusted significance was considered an indicator of statistical robustness but was not used as the exclusive criterion for candidate prioritization. Instead, candidate selection was based on an integrated strategy incorporating differential analysis, multivariate analysis, pathway-level interpretation, machine-learning-based feature evaluation, biological plausibility, and feasibility of targeted assessment.

Random Forest (RF) analysis was subsequently performed to identify metabolites contributing to discrimination between CAVS patients and controls. The metabolomic dataset was arranged with samples as observations and metabolites as predictor variables. Before model construction, metabolite features were log-transformed and scaled. A RF classifier was constructed using 1,000 decision trees, and model performance was evaluated using the out-of-bag (OOB) error estimate. Ten-fold cross-validation was additionally performed to evaluate classification stability. Feature importance was assessed using mean decrease accuracy (MDA), and metabolites were ranked according to their contribution to classification performance. The RF analysis was used primarily for candidate feature prioritization rather than development of a clinically applicable predictive model. Importantly, serotonin was not selected solely based on individual metabolite ranking but through integration of pathway-level evidence, biological plausibility, and independent targeted assessment feasibility.

Metabolic signals identified from the integrated analytical strategy were further evaluated according to pathway relevance. Because pathway enrichment analysis reflects metabolite annotation and pathway-level association rather than direct metabolic flux measurement, enriched pathways were interpreted as biological signals requiring further validation. A biologically plausible candidate derived from the discovery-phase pathway-level annotation was subsequently evaluated in an independent cohort (n = 303) using a targeted ELISA assay.

Pathway enrichment analysis was performed using MetaboAnalyst (https://www.metaboanalyst.ca) and Kyoto Encyclopedia of Genes and Genomes (KEGG) database annotation (https://www.kegg.jp).

### 2.6 Serum serotonin detection

Serum serotonin levels were quantified using a commercial ELISA kit (E-EL-0033, Elabscience, Wuhan, China) according to the manufacturer’s instructions. Samples were appropriately diluted based on the standard curves. Optical density (OD) at 450 nm was measured with an automated microplate reader, and serum serotonin concentrations were calculated by interpolation. Serum samples were coded before ELISA analysis, and laboratory personnel performing serotonin measurements were blinded to clinical group assignments to minimize potential measurement bias. All samples were analyzed in triplicate, and mean values were used for statistical analyses to reduce analytical variability.

Because the majority of circulating serotonin is stored in platelets, pre-analytical variables were carefully controlled. Samples were allowed to clot for exactly 30 min at room temperature, then centrifuged at 3000 rpm for 15 min at 4 °C. Samples with visible hemolysis were excluded. PLT was measured routinely and considered as a covariate in the statistical analysis. Freeze-thaw cycles were limited to one before ELISA. The ELISA assay showed intra-assay and inter-assay coefficients of variation of <8% and <12%, respectively, according to the manufacturer’s data.

### 2.7 Statistical analysis

Statistical analyses were conducted using SPSS 29.0 (SPSS Inc., Chicago, IL, USA), R software version 4.4.1 (R Foundation for Statistical Computing, Vienna, Austria), and GraphPad Prism 10 (GraphPad Software Inc., San Diego, CA, USA). The completeness of all variables included in the primary analyses was assessed before statistical modeling. No missing values were identified for variables used in the logistic regression models, subgroup analyses, smooth curve fitting, or ROC analyses. Therefore, these analyses were performed using complete-case datasets without imputation. Variables with missing values that were not included in the primary models were analyzed using available-case data when applicable.

Continuous variables were assessed for normality and homogeneity of variance. Normally distributed data are expressed as mean ± standard deviation (SD) and were compared using one-way ANOVA; non-normally distributed data are presented as median (interquartile range) and analyzed using the Kruskal–Wallis test with Bonferroni correction. Categorical variables are summarized as frequencies (percentages) and compared by Chi-square test or Fisher’s exact test, with Bonferroni adjustment. The association between non-normally distributed variables was evaluated using Spearman’s rank correlation.

To examine the relationship between serotonin and CAVS, univariate logistic regression was performed, followed by multivariable logistic regression adjusting for potential confounders to evaluate whether serotonin remained associated with CAVS after covariate adjustment. The dependent variable in all logistic models was the presence of CAVS (control vs. any CAVS). Age and sex were considered prespecified core covariates and were forced into all multivariable models regardless of their univariate significance. Other covariates were selected based on clinical relevance and their associations with CAVS in univariate analyses. Medication variables (beta-blockers, CCBs, diuretics, hypoglycemic agents) were considered but not included in the final model because their relationships with CAVS may reflect underlying cardiovascular comorbidities and treatment indications. Multicollinearity was assessed using variance inflation factors (VIF). The nonlinear association between serum serotonin levels and the probability of CAVS presence was evaluated using smooth curve fitting based on generalized additive models (GAMs), with adjustment for age, sex, and other clinically relevant covariates.

Subgroup analyses were conducted to assess the robustness and potential effect modification of the association between serum serotonin and CAVS. Participants were stratified according to clinically relevant or median-based cutoff values of age, PLT, high-density lipoprotein cholesterol (HDL-C), low-density lipoprotein cholesterol (LDL-C), triglycerides (TG), Lp(a), free fatty acids (FFA), and phospholipase A2 (PLA2). Within each subgroup, multivariable logistic regression models were fitted to estimate adjusted odds ratios (ORs) and 95% confidence intervals (CIs). The same covariates used in the primary multivariable model were applied for adjustment, except that the corresponding stratification variable was not included in the adjustment model to avoid overadjustment and potential collinearity. Interaction terms between serum serotonin and each stratification variable were introduced into the regression models to evaluate potential effect modification. PLT was included as a covariate in the multivariable regression models because circulating serotonin concentrations are strongly influenced by platelet storage and release.

Diagnostic performance of serotonin for CAVS presence was evaluated using receiver operating characteristic (ROC) curve analysis. Optimal cutoff values were determined by maximizing Youden’s index (J = sensitivity + specificity −1). The stability of the optimal cutoff value was assessed using bootstrap resampling with 2,000 repetitions. The incremental discriminatory value of serum serotonin was evaluated by comparing nested logistic regression models using DeLong’s test for differences in AUC. Model calibration was assessed using calibration plots, Brier score, calibration intercept, calibration slope, and the unreliability test. The events-per-variable ratio for the final multivariable models exceeded the commonly recommended threshold of 10, although this metric does not substitute for an *a priori* sample size calculation. Continuous net reclassification improvement (NRI) and integrated discrimination improvement (IDI) were calculated to evaluate the incremental value of adding serotonin to clinical models. Bootstrap resampling with 2,000 repetitions was performed to estimate confidence intervals for NRI and IDI. A two-sided P < 0.05 was considered statistically significant.

## 3 Results

### 3.1 Baseline characteristics of the discovery cohort

In the discovery cohort, 30 patients with severe CAVS and 30 age- and sex-matched controls were initially enrolled. One participant from each group was excluded due to inadequate blood sample quality, resulting in 29 patients and 29 controls included for metabolomic screening analysis. As summarized in Table 1, several clinical and biochemical parameters differed between the two groups. Compared with controls, patients with severe CAVS showed lower hemoglobin levels and significant differences in several metabolic and inflammatory parameters, including TC, LDL-C, TG, Lp(a), PLA2, and erythrocyte sedimentation rate (ESR). These differences indicate that the discovery cohort exhibited baseline metabolic heterogeneity, which is an important consideration when interpreting exploratory metabolomic findings.

**TABLE 1:** The baseline characteristics of participants in the discovery cohort.

Variables	Control group (N = 29)	CAVS group (N = 29)	P value
Age (years old)	66.8 ± 5.1	69.7 ± 7.5	0.096
Male (n, %)	17 (58.6%)	13 (44.8%)	0.293
SBP (mmHg)	132.0 ± 15.9	130.3 ± 25.4	0.758
DBP (mmHg)	74.1 ± 12.9	73.9 ± 12.4	0.951
BMI (kg/m^2^)	25.3 ± 3.0	24.2 ± 3.7	0.241
History of smoking (n, %)	15 (51.7%)	9 (31.0%)	0.110
History of hypertension (n, %)	18 (62.1%)	14 (48.3%)	0.291
History of diabetes (n, %)	6 (20.7%)	10 (34.5%)	0.240
White blood cell (×10^9^/L)	6.4 ± 1.8	6.4 ± 2.5	0.909
Red blood cell (×10^12^/L)	4.5 ± 0.4	4.3 ± 0.5	0.124
Hemoglobin (g/L)	137.4 ± 11.6	130.0 ± 16.6	0.055
Platelet (×10^9^/L)	214.7 ± 55.6	218.2 ± 64.8	0.825
Neutrophil (×10^9^/L)	3.7 ± 1.4	4.1 ± 2.6	0.508
hs-CRP (mg/L)	1.2 (0.7, 1.5)	1.4 (0.6, 7.3)	0.581
ESR (mm/h)	14.0 (6.2, 26.0)	33.0 (15.8, 52.0)	0.042
ALT (U/L)	20.0 (17.0, 26.0)	14.0 (12.0, 25.0)	0.057
AST (U/L)	23.6 ± 11.5	23.4 ± 15.3	0.954
Total cholesterol (mmol/L)	3.9 ± 1.2	4.5 ± 1.2	0.043
HDL-C (mmol/L)	1.2 ± 0.2	1.3 ± 0.3	0.095
LDL-C (mmol/L)	2.2 ± 1.0	2.8 ± 1.1	0.036
Triglycerides (mmol/L)	1.5 ± 0.7	1.1 ± 0.4	0.028
Lp(a) (nmol/L)	29.4 (12.3, 60.7)	111.8 (18.6, 167.7)	0.017
Homocysteine (µmol/L)	11.7 (9.2, 13.6)	11.7 (9.8, 14.4)	0.924
FFA (µmol/L)	50.1 ± 24.2	50.8 ± 22.1	0.921
PLA2 (U/L)	448.5 ± 123.5	542.2 ± 147.9	0.013
FBG (mmol/L)	5.8 ± 1.4	5.9 ± 2.1	0.905
Creatinine (µmol/L)	65.0 (57.0, 75.0)	69.0 (58.0, 83.0)	0.508
Uric acid (µmol/L)	307.8 ± 66.9	323.8 ± 93.9	0.461
eGFR (mL/min/1.73 m^2^)	91.2 ± 16.4	86.1 ± 14.7	0.217

Continuous variables with a normal distribution are expressed as mean ± SD; otherwise, they are presented as median (interquartile range) [M (Q1–Q3)]. CAVS, calcific aortic valve stenosis; ALT, alanine aminotransferase; AST, aspartate aminotransferase; BMI, body mass index; DBP, diastolic blood pressure; eGFR, estimated glomerular filtration rate; ESR, erythrocyte sedimentation rate; FBG, fasting blood glucose; FFA, free fatty acids; HDL-C, high-density lipoprotein cholesterol; hs-CRP, high-sensitivity C-reactive protein; LDL-C, low-density lipoprotein cholesterol; Lp(a), Lipoprotein(a); PLA2, phospholipase A2; SBP, systolic blood pressure.

### 3.2 Metabolic profiling and differential metabolite identification

Following raw data processing and feature detection, a total of 6,552 and 4,048 precursor molecules were detected in positive and negative ion modes, respectively. Quality control was assessed by Pearson’s correlation analysis of all QC samples. The high correlation coefficients observed among QC samples (Supplementary Figure S1) indicated good analytical reproducibility and instrument stability.

Principal component analysis (PCA) revealed a tendency toward separation between the CAVS and control groups (Figure 1A), while partial least squares discriminant analysis (PLS-DA) showed separation between the two groups (Figure 1B). The model showed strong separation performance, with R^2^ = 0.93 and Q^2^ = 0.83 (Supplementary Table S1). Permutation testing further supported model robustness, with an *R* ^2^ intercept of 0.75 and a Q^2^ intercept of −0.36, and the permutation test did not indicate significant overfitting (permutation P > 0.05) (Figure 1C).

**FIGURE 1:**
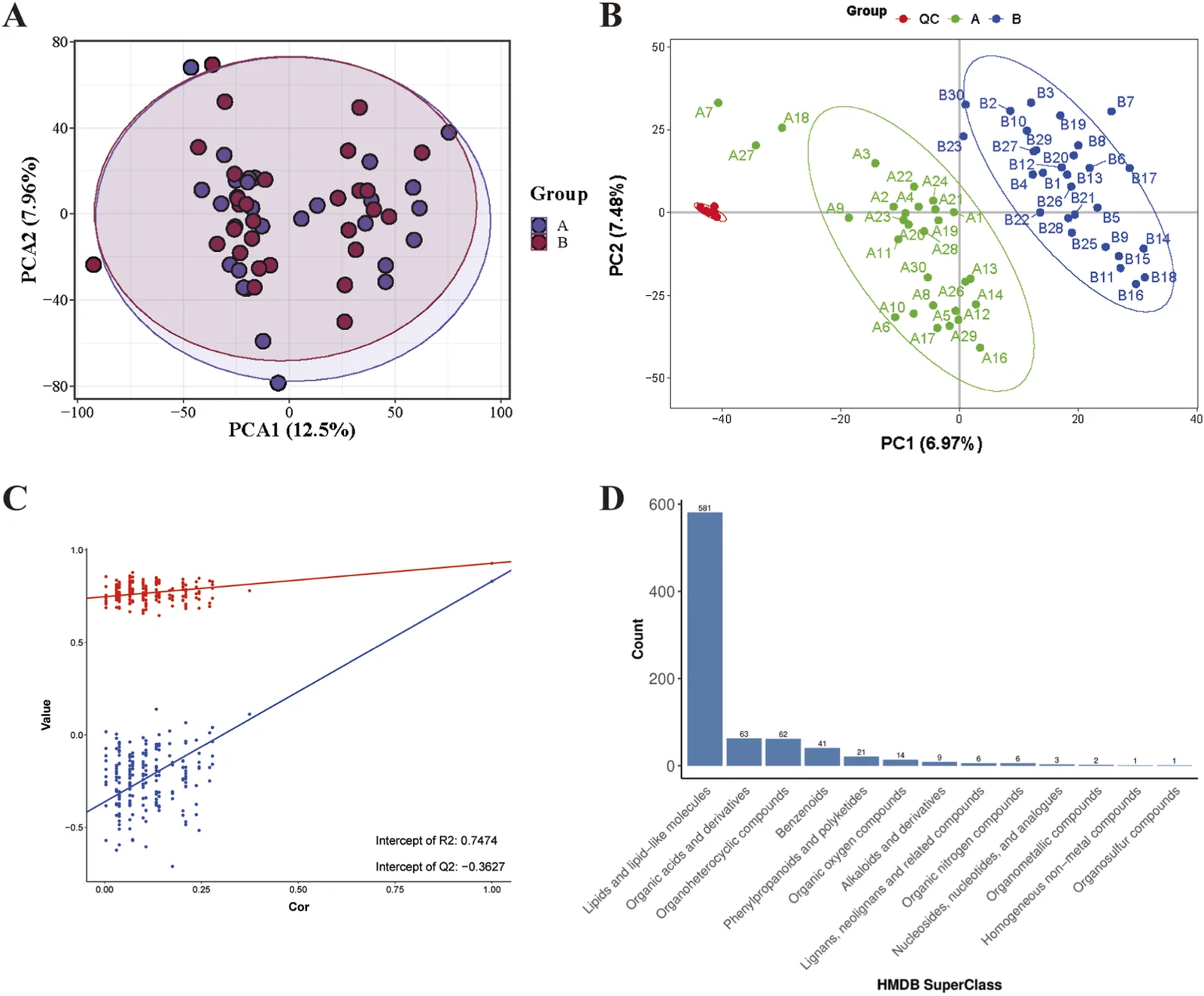
Metabolomic profiling of serum samples was performed in patients with CAVS and age- and sex-matched controls. **(A)** Principal component analysis (PCA) score plot. **(B)** Partial least squares-discriminant analysis (PLS-DA) score plot. **(C)** Permutation test. **(D)** Classification of identified metabolites based on the HMDB. HMDB, Human Metabolome Database; PC, principal component; PCA, principal component analysis.

Metabolite annotation was performed using the KEGG and HMDB databases. HMDB-based categorization (Figure 1D) showed that “Lipids and lipid-like molecules” were the most abundant class.

### 3.3 Pathway enrichment identifies serotonin-related metabolic alterations

From the 9,777 metabolic ions detected, applying the criteria of |fold change| > 1.2, P < 0.05, and VIP >1, we identified 707 differentially abundant metabolic ions (Figure 2A). To control false discovery in this high-dimensional dataset, we applied FDR correction (Benjamini–Hochberg, q < 0.05) to all 9,777 detected ions; after correction, 122 metabolites remained significant (q < 0.05), supporting the presence of statistically significant metabolic alterations in the discovery cohort.

**FIGURE 2:**
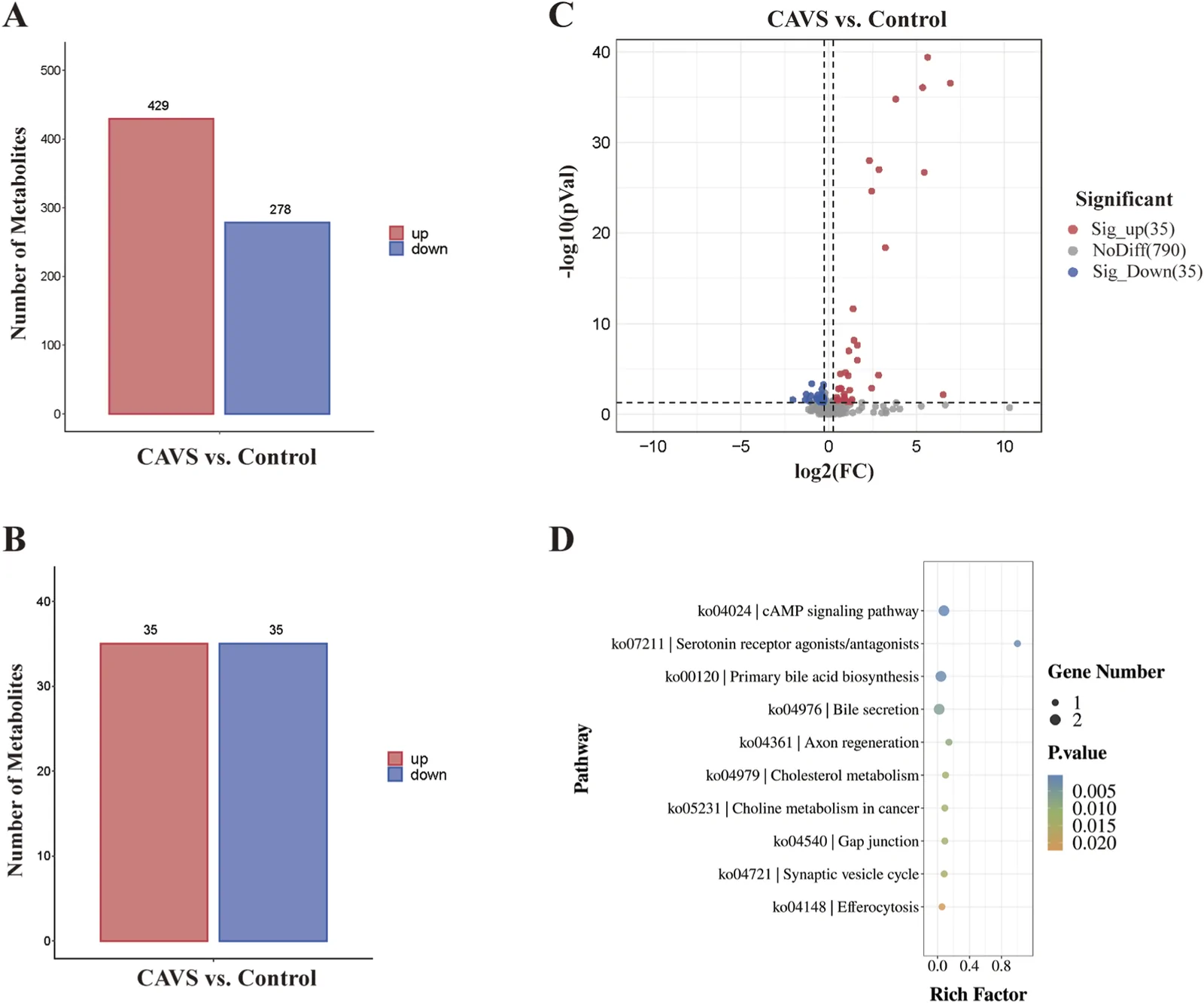
Differential metabolites and enriched pathways in CAVS. **(A)** The number of significantly altered metabolic ions. Screening parameters were P < 0.05, fold change >1.2, and VIP >1 from PLS-DA analysis. **(B)** The number of annotated differential secondary metabolites. Screening parameters were P < 0.05, |fold change| > 1.2, and a variable importance in projection (VIP) score from PLS-DA analysis. **(C)** Volcano plot of the 70 differential secondary metabolites. Screening parameters were P value <0.05, |fold change| > 1.2, and VIP >1. Red dots represent upregulated metabolites, blue dots represent downregulated metabolites, and gray dots represent metabolites with non-significant changes. **(D)** The top ten enriched KEGG pathways based on metabolite annotation.

Of the 707 differentially abundant ions, 70 secondary metabolites were putatively annotated, with 35 upregulated and 35 downregulated (Figure 2B). KEGG pathway enrichment analysis identified several enriched pathways, including serotonin-related signaling pathways, bile acid metabolism, and lipid-associated pathways (Figure 2D). Detailed enrichment parameters, including pathway size, matched metabolite number, enrichment factor, P value, and FDR-adjusted q value, are provided in Supplementary Table S2.

The serotonin-related pathway emerged as one of the enriched pathways in KEGG compound enrichment analysis (enrichment P = 0.001246, FDR-adjusted q = 0.014270), suggesting a sparse pathway-level metabolic association with CAVS. Because only one metabolite matched this pathway, the finding was considered hypothesis-generating rather than evidence of direct serotonin pathway activation. Importantly, serotonin itself did not reach FDR significance (q = 0.48) in the discovery metabolomic analysis. Therefore, serotonin was selected for targeted assessment based on the integration of pathway-level evidence, biological plausibility, and existing mechanistic evidence, rather than as a directly identified differential metabolite.

### 3.4 Selection and independent targeted assessment of serotonin as a candidate biomarker

Random Forest (RF) analysis was performed as an exploratory machine-learning approach to rank metabolites according to their contribution to group discrimination. The RF model achieved complete classification separation within the discovery cohort, with an out-of-bag (OOB) error rate of 0% (OOB accuracy = 100%). Ten-fold cross-validation further showed classification accuracies ranging from 93.0% to 100% across different mtry parameters (Supplementary Table S3). However, given the limited sample size and high dimensionality of untargeted metabolomic data, these results were interpreted cautiously and the RF analysis was used primarily for feature prioritization rather than development of a clinically applicable predictive model.

Feature importance ranking based on mean decrease accuracy identified several metabolites with higher contribution to group discrimination, including 25-hydroxycholesterol, octadecanamide, oleamide, artocarpin, and 2-hexenoylcarnitine (Figure 3A; Supplementary Table S4). Indoleacrylic acid was also identified among the metabolites contributing to group discrimination and represents a microbiota-derived indole metabolite involved in tryptophan-related metabolism.

**FIGURE 3:**
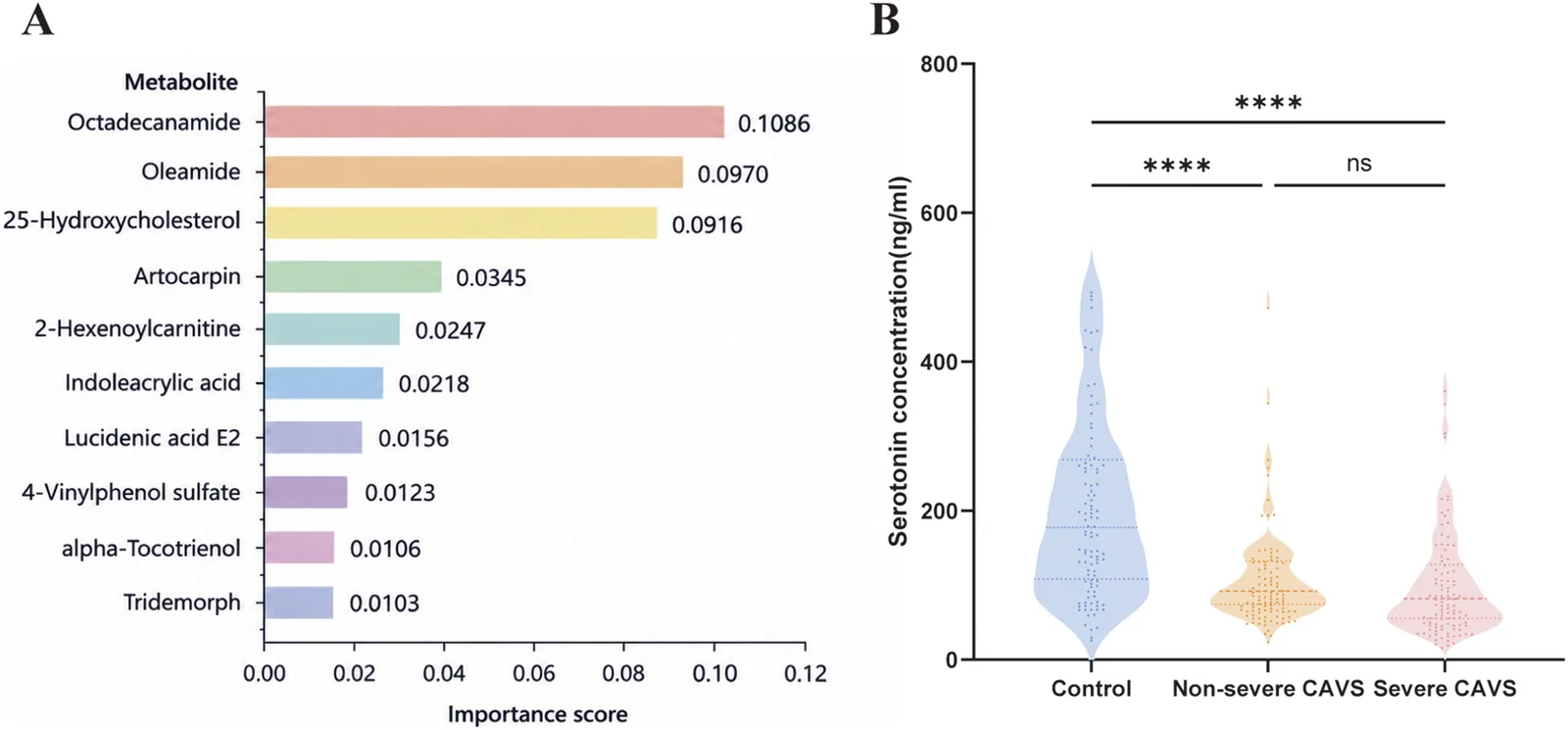
Machine-learning prioritization of discriminatory metabolites and independent targeted assessment of serum serotonin levels in CAVS. **(A)** Random Forest analysis showing the relative importance of metabolites for discrimination between CAVS patients and controls. **(B)** Serum serotonin concentrations in the independent targeted assessment cohort.

Importantly, serotonin itself was not among the highest-ranked individual metabolites in the RF analysis. Therefore, serotonin was not prioritized based on machine-learning ranking alone, but rather selected through integration of pathway-level evidence, biological plausibility, and feasibility of targeted assessment in an independent targeted assessment cohort.

### 3.5 Baseline characteristics of the independent targeted assessment cohort

For the targeted assessment cohort, 303 participants were enrolled, comprising 101 controls, 101 patients with non-severe CAVS, and 101 patients with severe CAVS. Demographic characteristics, including age and sex, were broadly comparable across the three groups. However, significant differences between groups were observed in several medication histories (β-blockers, CCBs, diuretics, and hypoglycemic agents) and laboratory parameters, including hemoglobin, PLT, ESR, triglycerides, Lp(a), PLA2, creatinine, uric acid, and eGFR (all detailed in Table 2).

**TABLE 2:** The baseline characteristics of participants in the independent targeted assessment cohort.

Variables	Control group (N = 101)	Non-severe CAVS group (N = 101)	Severe CAVS group (N = 101)	P value
Age (years old)	69.7 ± 6.7	71.7 ± 10.3	71.2 ± 9.0	0.238
Male (n, %)	58 (57.4%)	59 (58.4%)	60 (59.4%)	0.960
SBP (mmHg)	130.8 ± 15.0	134.7 ± 22.7	131.9 ± 21.3	0.371
DBP (mmHg)	75.4 ± 10.0	74.0 ± 12.2	73.2 ± 12.0	0.383
BMI (kg/m^2^)	25.2 ± 3.5	24.2 ± 3.5	24.9 ± 4.1	0.195
History of smoking (n, %)	28 (27.7%)	38 (37.6%)	34 (33.7%)	0.322
History of hypertension (n, %)	58 (57.4%)	70 (69.3%)	60 (59.4%)	0.176
History of diabetes (n, %)	27 (26.7%)	32 (31.7%)	38 (37.6%)	0.252
Medical history of ACEI/ARB (n, %)	43 (42.6%)	38 (37.6%)	38 (37.6%)	0.708
Medical history of β-blockers (n, %)	46 (45.5%)	48 (47.5%)	64 (63.4%)	**0.021**
Medical history of CCBs (n, %)	20 (19.8%)	45 (44.6%)	28 (27.7%)	**< 0.001**
Medical history of diuretics (n, %)	19 (18.8%)	45 (44.6%)	73 (72.3%)	**< 0.001**
Medical history of hypoglycemic agents (n, %)	28 (27.7%)	43 (42.6%)	52 (51.5%)	**0.002**
Medical history of anti-platelet drugs (n, %)	56 (55.4%)	55 (54.5%)	62 (61.4%)	0.560
Medical history of statin (n, %)	60 (59.4%)	62 (61.4%)	71 (70.3%)	0.230
White blood cell (×10^9^/L)	6.6 ± 2.0	6.5 ± 2.5	6.3 ± 1.9	0.702
Red blood cell (×10^12^/L)	4.6 ± 3.0	4.0 ± 0.7	4.3 ± 0.6	0.064
Hemoglobin (g/L)	133.1 ± 16.1	122.8 ± 22.6	129.6 ± 17.3	**< 0.001**
Platelet (×10^9^/L)	214.2 ± 55.0	192.0 ± 64.8	199.4 ± 55.1	**0.024**
Neutrophil (×10^9^/L)	4.1 ± 1.7	4.1 ± 2.0	3.9 ± 1.4	0.612
hs-CRP (mg/L)	1.5 (1.1, 1.7)	1.5 (1.5, 1.5)	1.5 (1.5, 1.5)	0.119
ESR (mm/h)	17.0 (10.0, 17.0)	17.0 (17.0, 17.0)	17.0 (17.0, 18.0)	**0.004**
ALT (U/L)	17.0 (14.0, 23.0)	15.0 (11.0, 21.0)	17.0 (12.0, 25.0)	0.211
AST (U/L)	21.7 ± 13.5	22.9 ± 14.2	24.3 ± 15.2	0.433
Total cholesterol (mmol/L)	4.1 ± 0.9	4.2 ± 1.3	4.3 ± 1.0	0.290
HDL-C (mmol/L)	1.2 ± 0.3	1.2 ± 0.3	1.2 ± 0.3	0.358
LDL-C (mmol/L)	2.4 ± 0.7	2.5 ± 0.9	2.6 ± 0.9	0.325
Triglycerides (mmol/L)	1.5 ± 0.8	1.3 ± 0.6	1.2 ± 0.6	**0.007**
Lp(a) (nmol/L)	62.5 (29.7, 62.5)	62.5 (38.2, 110.8)	62.5 (42.0, 145.2)	**0.034**
Homocysteine (µmol/L)	13.7 ± 8.1	14.7 ± 7.1	15.6 ± 7.0	0.204
FFA (µmol/L)	51.2 ± 19.7	57.3 ± 24.5	58.5 ± 24.7	0.056
PLA2 (U/L)	472.0 ± 100.4	522.3 ± 135.5	520.8 ± 129.2	**0.005**
FBG (mmol/L)	5.8 ± 1.3	5.9 ± 1.8	6.1 ± 1.8	0.451
Creatinine (µmol/L)	73.2 ± 21.1	90.8 ± 74.6	81.5 ± 28.8	**0.034**
Uric acid (µmol/L)	304.7 ± 80.0	318.7 ± 120.4	344.6 ± 116.4	**0.029**
eGFR (mL/min/1.73 m^2^)	88.7 ± 17.7	79.6 ± 22.0	81.7 ± 19.3	**0.003**

Continuous variables with a normal distribution are expressed as mean ± SD; otherwise, they are presented as median (interquartile range) [M (Q1–Q3)]. CAVS, calcific aortic valve stenosis; ALT, alanine aminotransferase; AST, aspartate aminotransferase; BMI, body mass index; DBP, diastolic blood pressure; eGFR, estimated glomerular filtration rate; ESR, erythrocyte sedimentation rate; FBG, fasting blood glucose; FFA, free fatty acids; HDL-C, high-density lipoprotein cholesterol; hs-CRP, high-sensitivity C-reactive protein; LDL-C, low-density lipoprotein cholesterol; Lp(a), Lipoprotein(a); PLA2, phospholipase A2; SBP, systolic blood pressure. Bold values indicate statistically significant differences (P < 0.05).

### 3.6 Lower serum serotonin levels are associated with CAVS presence

Serotonin levels were significantly lower in both non-severe and severe CAVS groups compared with controls (control: 177.6 ± 68.7 ng/mL; non-severe: 92.1 ± 17.5 ng/mL; severe: 82.1 ± 26.5 ng/mL; both P < 0.001 vs. control), whereas no significant difference was observed between the two CAVS groups (Figure 3B). These findings indicate that circulating serotonin levels were associated with the presence of CAVS but did not show a significant gradient according to disease severity. Therefore, serum serotonin was interpreted as a potential marker of CAVS presence rather than a marker of disease progression.

### 3.7 Serotonin shows an independent association with CAVS after multivariable adjustment

Univariate logistic regression identified multiple significant correlates of CAVS, including hematological parameters (red blood cell count, hemoglobin, and PLT), lipid-related indices (triglycerides, Lp(a), FFA, and PLA2), renal function markers (creatinine, uric acid, and eGFR), and medication history (calcium channel blockers, diuretics, and hypoglycemic agents) (Table 3). Given the collinearity between red blood cell count and hemoglobin, only hemoglobin was retained in the subsequent multivariable model. Medication-related variables were not entered into the final model, because they may reflect underlying cardiovascular disease management and treatment status rather than independent confounders. Therefore, their potential influence should be interpreted cautiously and should be further evaluated in dedicated longitudinal studies.

**TABLE 3:** Univariate and multivariable logistic regression analyses of factors associated with CAVS.

Variable	Univariate analysis		Multivariable analysis	
	OR (95% CI)	P value	OR (95% CI)	P value
Male	0.941 (0.580∼1.526)	0.805	0.882 (0.396∼1.965)	0.7595
Age	1.023 (0.996∼1.052)	0.101	1.010 (0.969∼1.054)	0.627
SBP	1.006 (0.994∼1.019)	0.313	​	​
DBP	0.986 (0.966∼1.007)	0.196	​	​
BMI	0.955 (0.896∼1.019)	0.166	​	​
History of smoking	1.444 (0.857∼2.434)	0.168	​	​
History of hypertension	1.339 (0.821∼2.181)	0.242	​	​
History of diabetes	1.453 (0.858∼2.463)	0.165	​	​
Medical history of ACEI/ARB	0.814 (0.500∼1.323)	0.406	​	​
Medical history of β-blockers	1.488 (0.921∼2.404)	0.105	​	​
Medical history of CCBs	2.292 (1.300∼4.042)	0.004	​	​
Medical history of diuretics	6.063 (3.421∼10.743)	<0.001	​	​
Medical history of hypoglycemic agents	2.315 (1.382∼3.878)	0.001	​	​
Medical history of anti-platelet drugs	1.106 (0.683∼1.791)	0.682	​	​
Medical history of statins	1.317 (0.805∼2.155)	0.273	​	​
White blood cell	0.963 (0.864∼1.075)	0.505	​	​
Red blood cell	0.586 (0.385∼0.891)	0.012	​	​
Hemoglobin	0.980 (0.967∼0.994)	0.004	0.986 (0.967∼1.006)	0.1647
Platelet	0.995 (0.991∼0.999)	0.011	**0.992 (0.987∼0.998)**	**0.0049**
Neutrophil	0.971 (0.847∼1.114)	0.677	​	​
hs-CRP	1.013 (0.991∼1.035)	0.250	​	​
ESR	1.032 (1.008∼1.056)	0.007	​	​
ALT	1.000 (0.989∼1.012)	0.953	​	​
AST	1.010 (0.991∼1.030)	0.290	​	​
Total cholesterol	1.186 (0.944∼1.490)	0.142	​	​
HDL-C	1.435 (0.638∼3.225)	0.382	​	​
LDL-C	1.220 (0.908∼1.638)	0.187	​	​
Triglycerides	0.591 (0.414∼0.844)	0.004	**0.618 (0.403∼0.949)**	**0.0277**
Lp(a)	1.004 (1.001∼1.008)	0.019	**1.009 (1.004∼1.014)**	**< 0.001**
Homocysteine	1.029 (0.992∼1.069)	0.129	​	​
FFA	1.013 (1.002∼1.025)	0.019	**1.019 (1.002∼1.035)**	**0.028**
PLA2	1.004 (1.001∼1.006)	0.001	**1.005 (1.002∼1.008)**	**0.0014**
FBG	1.096 (0.936∼1.283)	0.252	​	​
Creatinine	1.013 (1.003∼1.024)	0.015	1.006 (0.993∼1.019)	0.3576
Uric acid	1.003 (1.000∼1.005)	0.043	1.001 (0.997∼1.004)	0.6075
eGFR	0.978 (0.965∼0.991)	0.001	0.982 (0.958∼1.006)	0.1457
Serotonin	0.989 (0.985∼0.992)	<0.001	**0.985 (0.981∼0.990)**	**< 0.001**

CAVS, calcific aortic valve stenosis; ALT, alanine aminotransferase; AST, aspartate aminotransferase; BMI, body mass index; hs-CRP, high-sensitivity C-reactive protein; DBP, diastolic blood pressure; eGFR, estimated glomerular filtration rate; ESR, erythrocyte sedimentation rate; FBG, fasting blood glucose; FFA, free fatty acids; HDL-C, high-density lipoprotein cholesterol; LDL-C, low-density lipoprotein cholesterol; Lp(a), Lipoprotein(a); PLA2, phospholipase A2; SBP, systolic blood pressure. Bold values indicate statistically significant differences (P < 0.05).

In a multivariable logistic regression model adjusting for age, sex, hemoglobin, PLT, creatinine, uric acid, eGFR, triglycerides, Lp(a), FFA, and PLA2, serum serotonin remained significantly associated with CAVS (OR = 0.985, 95% CI: 0.981–0.990, P < 0.001). Additional independent correlates included PLT (OR = 0.992, 95% CI: 0.987–0.998, P = 0.005), triglycerides (OR = 0.618, 95% CI: 0.403–0.949, P = 0.028), Lp(a) (OR = 1.009, 95% CI: 1.004–1.014, P < 0.001), FFA (OR = 1.019, 95% CI: 1.002–1.035, P = 0.028), and PLA2 (OR = 1.005, 95% CI: 1.002–1.008, P = 0.001) (Table 3). Smooth curve fitting using a generalized additive model demonstrated a significant inverse association between serum serotonin levels and the probability of CAVS presence, without significant evidence of non-linearity (P for overall <0.001; P for non-linearity = 0.136) (Figure 4).

**FIGURE 4:**
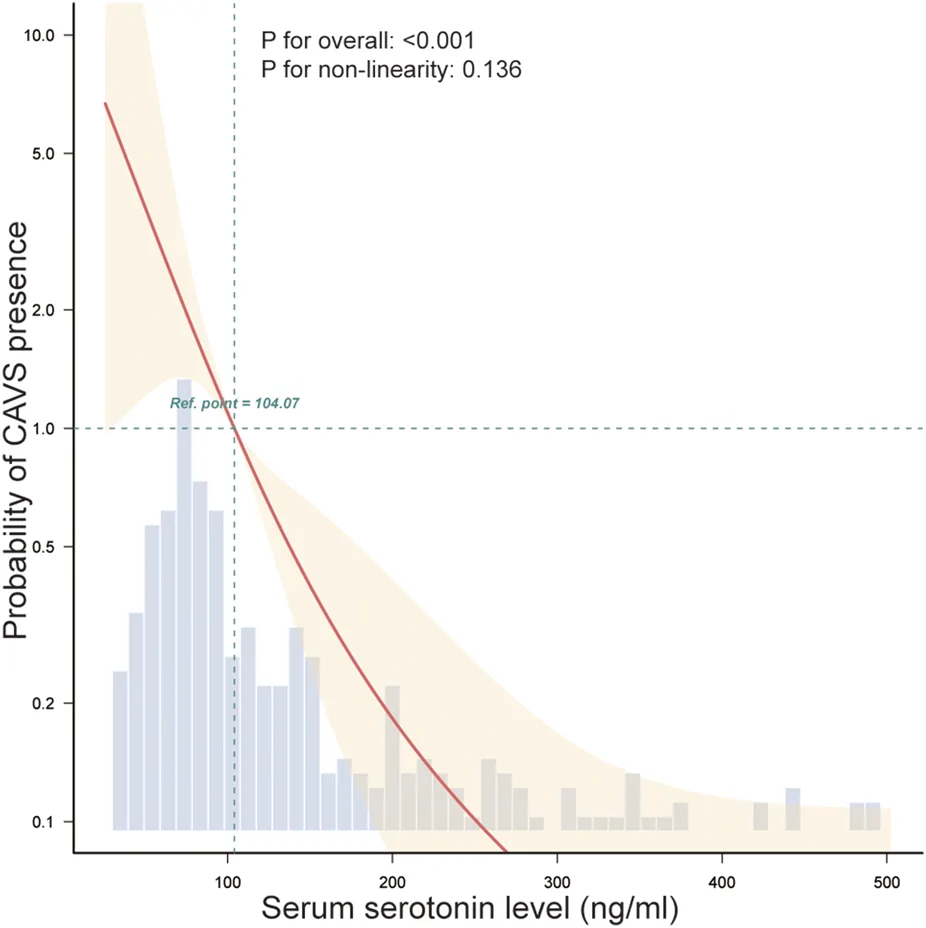
Association between serum serotonin levels and the probability of CAVS presence. Smooth curve fitting analysis was performed using a generalized additive model (GAM) adjusted for age, sex, hemoglobin, platelet count, triglycerides, PLA2, Lp(a), free fatty acids, creatinine, uric acid, and eGFR. The solid line represents the estimated probability of CAVS presence according to serum serotonin levels, and the shaded area represents the 95% confidence interval. The overall association was statistically significant (P for overall <0.001), whereas no significant evidence of non-linearity was observed (P for non-linearity = 0.136).

### 3.8 Subgroup analyses of the association between serum serotonin and CAVS

To evaluate the robustness of the inverse association between serum serotonin and CAVS, we performed subgroup analyses stratified by age, PLT, HDL-C, LDL-C, triglycerides, Lp(a), FFA, and PLA2, using median or clinically relevant cutoff values for each stratification variable (Figure 5).

**FIGURE 5:**
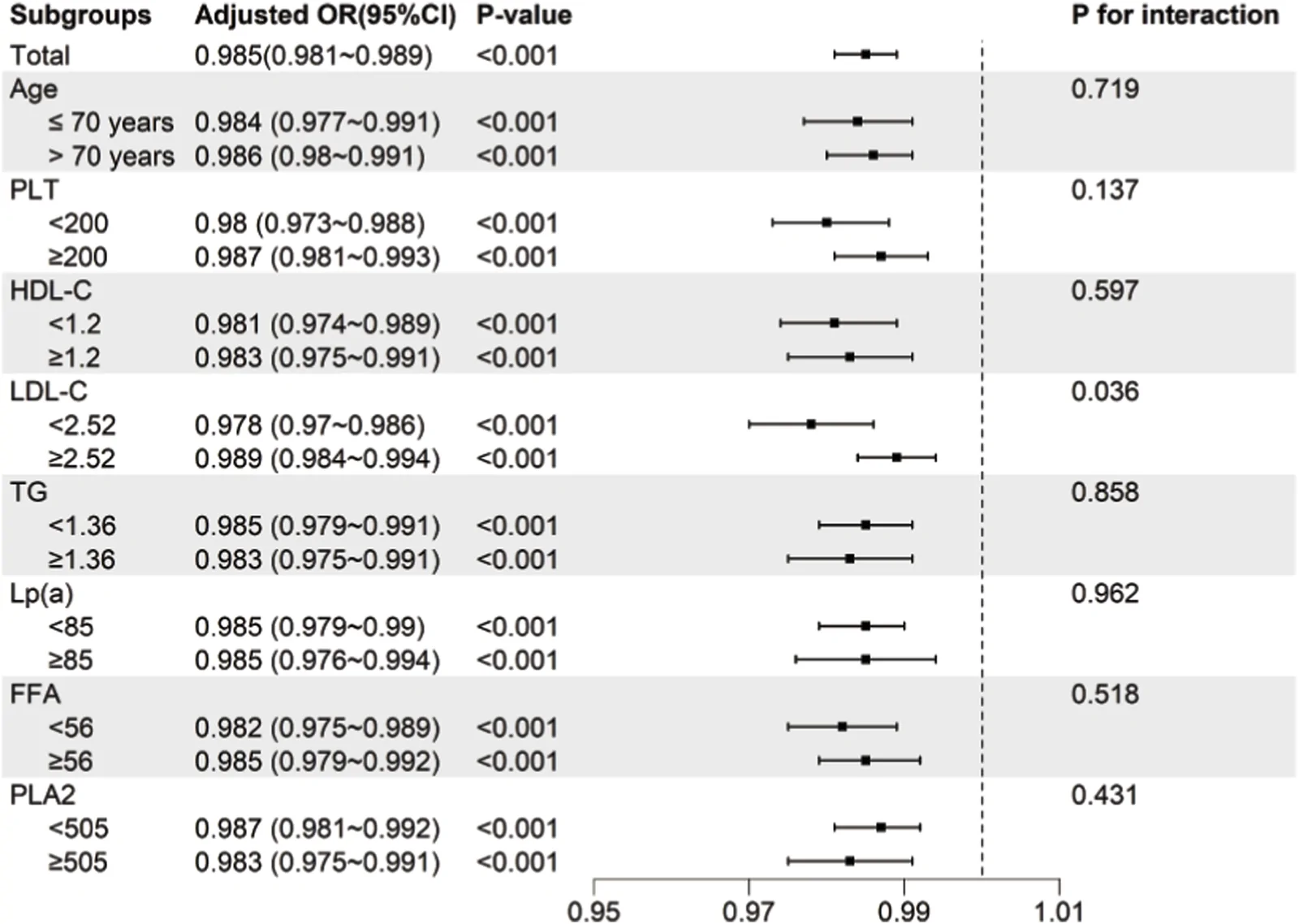
Subgroup analysis of the association between serum serotonin and CAVS. Forest plot showing the association between serum serotonin levels and the presence of CAVS across subgroups stratified by age, PLT, HDL-C, LDL-C, TG, Lp(a), FFA, and PLA2 levels. FFA, free fatty acids; HDL-C, high-density lipoprotein cholesterol; LDL-C, low-density lipoprotein cholesterol; Lp(a), Lipoprotein(a); PLA2, phospholipase A2; PLT, platelet count; TG, triglycerides.

The inverse association between serum serotonin and CAVS remained consistently significant across all predefined subgroups (all P < 0.001), with adjusted odds ratios ranging from 0.97 to 0.994. No significant interaction was observed for age (P for interaction = 0.719), PLT (P = 0.137), HDL-C (P = 0.597), triglycerides (P = 0.858), Lp(a) (P = 0.962), FFA (P = 0.518), or PLA2 (P = 0.431), indicating that the observed association was generally consistent across these clinically relevant subgroups.

Notably, a significant interaction was detected for LDL-C (P for interaction = 0.036). The inverse association appeared stronger among participants with LDL-C < 2.52 mmol/L (OR = 0.978, 95% CI: 0.97–0.986) compared with those with LDL-C ≥ 2.52 mmol/L (OR = 0.989, 95% CI: 0.984–0.994). However, given the exploratory nature of multiple interaction analyses and the absence of adjustment for multiple comparisons in the primary analysis, this finding should be interpreted as hypothesis-generating rather than evidence of a confirmed biological interaction.

### 3.9 Incremental discriminatory value of serum serotonin for CAVS

To evaluate the incremental discriminatory value of serum serotonin for CAVS presence, participants were categorized into controls and patients with CAVS (including both non-severe and severe cases). Three logistic regression models were constructed: Model one included serum serotonin alone; Model two incorporated conventional clinical variables including age, sex, hemoglobin, PLT, triglycerides, PLA2, Lp(a), free fatty acids, creatinine, uric acid, and eGFR; Model three was established by adding serum serotonin to Model 2.

Serum serotonin alone demonstrated moderate discriminatory ability for CAVS, with an area under the ROC curve (AUC) of 0.774 (95% CI: 0.717–0.831, P < 0.001). The optimal cutoff value determined by Youden’s index was 137.1 ng/mL, corresponding to a sensitivity of 77.7% and a specificity of 66.3%. Bootstrap resampling with 2,000 repetitions demonstrated that the optimal cutoff value remained stable, with a 95% percentile confidence interval of 106.6–175.9 ng/mL.

The clinical model (Model 2) yielded an AUC of 0.752 (95% CI: 0.697–0.807, P < 0.001). After incorporation of serum serotonin, Model three showed improved discriminatory performance, achieving an AUC of 0.865 (95% CI: 0.824–0.907, P < 0.001) (Figure 6). The increase in AUC compared with Model two was statistically significant according to DeLong’s test (P < 0.001). The addition of serum serotonin further improved model-based classification performance, with a continuous NRI of 0.911 (95% CI: 0.687 -1.119) and an IDI of 0.210 (95% CI: 0.153–0.263) (Table 4). Calibration assessment of Model three demonstrated good agreement between predicted and observed probabilities (Supplementary Figure S2). The model showed a Brier score of 0.137, a calibration intercept of approximately 0.000, a calibration slope of 1.000, and no evidence of calibration deviation (unreliability test P = 0.847). The events-per-variable ratio for the final multivariable model was 16.8, suggesting an acceptable ratio of observed events to model parameters, although this does not replace prespecified sample size estimation.

**FIGURE 6:**
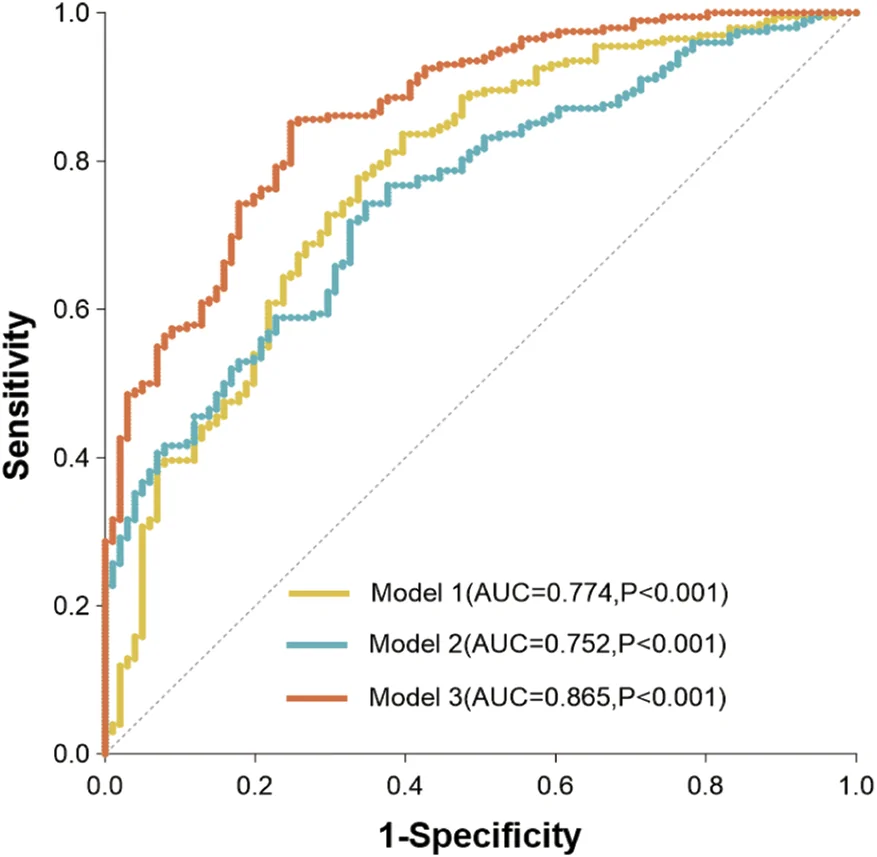
Incremental value of serum serotonin for improving discrimination of CAVS. Receiver operating characteristic (ROC) curve analysis of clinical models with and without serum serotonin. Model 1: serum serotonin alone; Model 2: conventional clinical variables including age, sex, hemoglobin, platelet count, triglycerides, PLA2, Lp(a), free fatty acids, creatinine, uric acid, and eGFR. Model three was established by adding serum serotonin to Model 2. AUC, area under the receiver operating characteristic curve; CAVS, calcific aortic valve stenosis.

**TABLE 4:** Incremental discriminatory value of serum serotonin for CAVS presence.

Discrimination for CAVS presence	Model 2	Model 3	P value
AUC	0.752 (0.697–0.807)	0.865 (0.824–0.907)	<0.001
NRI	Reference	0.911 (0.687–1.119)	<0.001
IDI	Reference	0.210 (0.153–0.263)	<0.001

Model 1: serum serotonin levels; Model 2: age, sex, hemoglobin, PLT, triglycerides, PLA2, Lp(a), FFA, creatinine, uric acid, eGFR; Model 3: Model two plus serum serotonin. CAVS, calcific aortic valve stenosis; ROC, receiver operating characteristic curve; AUC, area under the ROC curve.

Collectively, these findings indicate that serum serotonin provides additional discriminatory information beyond conventional clinical variables for discriminating individuals with and without CAVS. However, given the cross-sectional design, these findings should be interpreted as diagnostic association rather than evidence of predictive capability or causal contribution.

## 4 Discussion

This metabolomics study yielded several principal findings. First, systemic metabolic disturbances were evident in CAVS patients, with a serotonin-related metabolic annotation signal identified as a hypothesis-generating finding. Second, serum serotonin levels were significantly lower in CAVS patients than in controls, with no significant difference between non-severe and severe CAVS groups. Third, reduced serum serotonin was associated with CAVS presence after multivariable adjustment. Collectively, these support circulating serotonin as a biologically plausible candidate biomarker associated with CAVS, requiring prospective evaluation before clinical application.

Importantly, serotonin was not identified as the highest-ranked discriminatory metabolite in the RF analysis. Moreover, serotonin itself did not reach FDR significance in the discovery metabolomic analysis. Rather than representing a lead metabolite identified directly by differential abundance or machine-learning ranking, serotonin was selected based on the integration of pathway-level metabolic annotation, biological plausibility, prior mechanistic evidence, and feasibility of targeted assessment. In the present study, serotonin was selected because serotonin-related pathway signals were identified in KEGG enrichment analysis and because previous studies have established a potential relationship between serotonergic signaling, platelet biology, and valvular remodeling (Mekontso-Dessap et al., 2006; Bouchareb et al., 2019; Fong et al., 2021). However, only one metabolite was mapped to the serotonin-related pathway, and this pathway-level finding should therefore be regarded as sparse and hypothesis-generating rather than evidence of serotonergic system dysregulation. Nevertheless, this prioritization remains exploratory, and serotonin should be regarded as a candidate biomarker requiring independent confirmation rather than a definitive metabolic driver of CAVS.

Our findings further support the involvement of lipid-related metabolic alterations in CAVS, with “Lipids and lipid-like molecules” constituting the most altered metabolite class (Small et al., 2017). This is reinforced by the enrichment of pathways related to primary bile acid biosynthesis and cholesterol metabolism, indicating profound lipid homeostasis disruption. Dyslipidemia, particularly elevated LDL-C and Lp(a), is a well-established risk factor for CAVS progression (Smith et al., 2014; Tomova et al., 2020). Lp(a), a genetically influenced particle (Thanassoulis et al., 2013), mediates pathology largely via its cargo of oxidized phospholipids (OxPLs), which are delivered to valvular tissues and trigger pro-inflammatory and pro-osteogenic signaling in valvular interstitial cells (VICs) (Zheng et al., 2019). Furthermore, bile acids, acting as signaling molecules through receptors like the farnesoid X receptor (FXR) and the G protein-coupled bile acid receptor 5 (TGR5), can modulate inflammation and calcium metabolism—processes relevant to CAVS (Sun et al., 2019; Holeček, 2026). This is supported by evidence showing distinct bile acid profiles in calcified valves, correlating with disease severity (Zhang et al., 2025). However, these pathway findings should be interpreted as metabolic associations rather than direct evidence of altered pathway activity or causal involvement in valve calcification.

Serotonin is a biologically active monoamine with diverse central and peripheral functions. In peripheral tissues, serotonin is predominantly synthesized in the gastrointestinal tract and transported into platelets, where it is stored in dense granules and released upon platelet activation (Mekontso-Dessap et al., 2006). Because circulating serotonin concentrations are strongly influenced by platelet storage and sample processing conditions, serum serotonin measurements should be interpreted as an indicator of systemic serotonin homeostasis and platelet-related serotonin handling rather than a direct surrogate of tissue-level serotonergic activity. The potential involvement of platelet biology in CAVS has been suggested by alterations in platelet-related parameters observed in these patients (De Larochellière et al., 2019). Moreover, histological studies have reported platelet accumulation within diseased valves, suggesting a potential link between platelet biology and valvular remodeling; however, the functional consequences of this finding remain incompletely understood (Bouchareb et al., 2019).

The valvulopathic effects of excessive serotonin signaling, via 5-HT2B receptor stimulation on VICs, are well-documented in drug-induced and carcinoid-related valvulopathy (Fong et al., 2021). This established biological context makes the observed reduction in circulating serotonin in CAVS unexpected and highlights the complexity of interpreting systemic serotonin measurements in chronic valvular disease. Thus, serum serotonin does not directly represent the freely circulating serotonin pool but rather reflects the combined effects of platelet serotonin storage, activation, release dynamics, and systemic metabolic regulation. Although previous experimental studies suggest that platelet-derived serotonin may participate in local vascular and valvular remodeling, our study did not directly assess valvular serotonin content, platelet serotonin stores, serotonin transporter expression, or serotonergic receptor activity. Therefore, the present findings cannot determine whether reduced circulating serotonin reflects altered platelet handling, local serotonin utilization, compensatory metabolic adaptation, or secondary consequences of established CAVS.

Accordingly, the reduced serum serotonin observed in CAVS patients should not be interpreted as evidence that serotonin deficiency directly promotes valve calcification or that serotonin itself has a causal protective role against CAVS. Instead, it may represent altered serotonin homeostasis associated with CAVS, including changes in platelet serotonin handling, systemic metabolic adaptation, or disease-related physiological responses. Given the observational design of the present study, mechanistic interpretations remain speculative. Future studies incorporating platelet serotonin measurements, platelet activation assessment, and valve tissue analyses will be necessary to clarify the mechanisms underlying altered serotonin profiles in CAVS.

In addition to serotonin-related signals, our machine learning analysis identified indoleacrylic acid as one of the important discriminatory metabolites between patients with CAVS and controls. Indoleacrylic acid is a microbiota-derived indole metabolite originating from tryptophan metabolism and represents a distinct branch of tryptophan-related metabolic regulation rather than a direct downstream product of serotonin synthesis (Holeček, 2026). Together with serotonin-related pathway annotations identified in KEGG analysis, these findings suggest that broader tryptophan-related metabolic alterations may be associated with CAVS. However, KEGG enrichment reflects metabolite annotation-based pathway association and does not provide evidence of direct pathway activation, receptor stimulation, or altered metabolic flux. Furthermore, the RF analysis was performed primarily for candidate feature prioritization within the discovery cohort and should not be interpreted as a clinically applicable predictive model because of the limited sample size and high dimensionality of metabolomic data. Therefore, the contribution of individual metabolites identified by machine learning, including indoleacrylic acid, requires assessment in larger cohorts and complementary experimental studies. Future studies integrating targeted metabolomics, isotope tracing approaches, microbiome profiling, and experimental models will be necessary to determine how serotonin-related metabolic alterations relate to CAVS development.

The search for CAVS biomarkers has identified several candidates. Lipid-related markers include oxidized LDL, HDL, and Lp(a) (Smith et al., 2014; Sun et al., 2019; Tomova et al., 2020). Metabolites like gut microbiota-derived TMAO and immunometabolic markers like FBP1 have also been implicated (Li et al., 2022; Xu et al., 2025). Inflammatory cytokines from activated monocytes (e.g., RANTES) and non-coding RNAs (e.g., miR-17-5p) show diagnostic potential, as do emerging assessments of hemodynamic alterations (Zhang et al., 2022; Galeone et al., 2025). Compared with these candidates, circulating serotonin may provide complementary information associated with metabolic alterations observed in CAVS.

This study has notable strengths, including its multi-phase design combining unbiased discovery with assessment in a sizable independent cohort, and the complementary use of traditional statistics and machine learning. Multivariable adjustment and generally consistent subgroup findings support the robustness of the observed association, although residual confounding cannot be excluded. Subgroup analyses further suggested that lipid-related factors may influence the magnitude of the association between serum serotonin and CAVS. Although a nominal interaction was observed for LDL-C (P for interaction = 0.036), this finding did not remain statistically significant after correction for multiple comparisons. Moreover, the inverse association between serotonin and CAVS remained significant in both LDL-C strata, suggesting that this observation should be interpreted as exploratory and hypothesis-generating rather than as a confirmed effect modification.

Several limitations should be acknowledged. First, this was a single-center observational study, and external multicenter validation is required. Second, no *a priori* sample size calculation was performed, and the cross-sectional design precludes causal inference. Third, the discovery-phase metabolomic comparison was not adjusted for baseline differences in lipid and inflammatory parameters, including cholesterol-related measures and inflammatory markers. Therefore, some pathway-level metabolic alterations identified in the discovery cohort may partly reflect residual confounding related to these baseline characteristics rather than CAVS-specific biological changes. Fourth, serum serotonin levels may be influenced by platelet storage and coagulation-related release; therefore, the findings should be interpreted as altered serotonin homeostasis rather than direct evidence of tissue-level serotonergic changes. Future studies incorporating platelet-poor plasma serotonin measurements, platelet serotonin content, and platelet-related functional assessments are needed to clarify the underlying mechanisms. Fifth, potential residual confounding cannot be excluded, including unmeasured serotonin-modulating medications (SSRIs, SNRIs, and MAOIs) and recent antibiotic exposure affecting gut microbiota-related metabolism. Finally, LC-MS and ELISA measurements were not directly compared in the same samples; therefore, this study does not represent formal cross-platform analytical validation.

In summary, this study integrates untargeted metabolomic screening, independent targeted assessment, and clinical model evaluation to evaluate circulating serotonin as a biologically prioritized, hypothesis-generating candidate associated with CAVS presence. Serum serotonin levels were lower in both non-severe and severe CAVS groups compared with controls, whereas no difference was observed between disease severity categories, suggesting that serotonin may primarily reflect the presence of CAVS rather than disease severity or progression. Its independent association after multivariable adjustment and improvement in diagnostic discrimination beyond conventional clinical factors support further investigation of serotonin as a potential adjunct marker for CAVS discrimination rather than a standalone diagnostic biomarker. Future studies incorporating standardized sample processing, platelet-related assessments, external validation cohorts, and longitudinal designs are needed to clarify the biological significance and clinical utility of serotonin-related alterations in CAVS.

## 5 Conclusion

This study demonstrates that reduced circulating serotonin is associated with CAVS. Serum serotonin levels were lower in both non-severe and severe CAVS compared with controls, but did not differ between severity groups, suggesting that circulating serotonin may primarily reflect CAVS presence rather than stenosis severity or disease progression. Multivariable logistic regression demonstrated that lower serum serotonin levels were independently associated with CAVS presence and improved discrimination beyond conventional clinical variables in this cohort. These findings support circulating serotonin as a candidate adjunct marker for CAVS discrimination rather than a standalone diagnostic biomarker. Further multicenter prospective studies and mechanistic investigations are required to evaluate its potential clinical utility and clarify the biological significance of altered serotonin profiles in CAVS.

## Data Availability

The raw data supporting the conclusions of this article will be made available by the authors, without undue reservation.
