# Supplementary material for: Circulating serotonin is associated with calcific aortic valve stenosis: a two-phase metabolomics study
Source: Front Pharmacol. 2026 Sep 23;17:1934998. doi: 10.3389/fphar.2026.1934998 (PMC13642326; doi:10.3389/fphar.2026.1934998)
Supplement: Supplementary file 1 [file Supplementaryfile1.docx]

Supplementary Material

# 1 Supplementary Figure

## Supplemental Figure 1. **Pearson’s correlation analysis of all QC samples.** Pearson’s correlation analysis of metabolite abundances across all quality control (QC) samples demonstrates high inter-sample correlation coefficients, confirming excellent analytical reproducibility and instrument stability throughout the LC-MS sequence.

**Supplementary Figure 2. Calibration plot of the multivariable logistic regression model incorporating serum serotonin for discrimination of CAVS.** The model included serum serotonin and clinically relevant covariates. The calibration curve demonstrated good agreement between predicted and observed probabilities, with a Brier score of 0.137, calibration intercept close to 0, calibration slope of 1.00, and low calibration error (Emax = 0.018).

**2	Supplementary Tables**

**Supplementary Table 1. Performance parameters of multivariate and machine learning models.**

| **Model** | **R²** | **Q²** | **Permutation P value (R²)** | **Permutation P value (Q²)** | **R² intercept** | **Q² intercept** |
| --- | --- | --- | --- | --- | --- | --- |
| PLS-DA | 0.93 | 0.83 | <0.001 | <0.001 | 0.75 | -0.36 |

R² represents the proportion of variance explained by the PLS-DA model, whereas Q² reflects predictive performance. Intercept values were obtained from permutation testing. A negative Q² intercept indicates that randomly permuted models showed poor predictive ability, supporting the robustness of the original model.

**Supplementary Table 2. Top 10 KEGG pathway enrichment analysis of differential metabolites in the discovery cohort.**

| Rank | Pathway ID | KEGG Pathway | Matched metabolites (n) | Pathway size | Rich factor | P value | FDR-adjusted Q value |
| --- | --- | --- | --- | --- | --- | --- | --- |
| 1 | ko04024 | cAMP signaling pathway | 2 | 8 | 0.0800 | 0.000401 | 0.012045 |
| 2 | ko07211 | Serotonin receptor agonists/antagonists | 1 | 8 | 1.0000 | 0.001246 | 0.014270 |
| 3 | ko00120 | Primary bile acid biosynthesis | 2 | 8 | 0.0426 | 0.001427 | 0.014270 |
| 4 | ko04976 | Bile secretion | 2 | 8 | 0.0206 | 0.005957 | 0.044678 |
| 5 | ko04361 | Axon regeneration | 1 | 8 | 0.1429 | 0.008690 | 0.049523 |
| 6 | ko04979 | Cholesterol metabolism | 1 | 8 | 0.1000 | 0.012394 | 0.049523 |
| 7 | ko04540 | Gap junction | 1 | 8 | 0.0909 | 0.013626 | 0.049523 |
| 8 | ko05231 | Choline metabolism in cancer | 1 | 8 | 0.0909 | 0.013626 | 0.049523 |
| 9 | ko04721 | Synaptic vesicle cycle | 1 | 8 | 0.0833 | 0.014857 | 0.049523 |
| 10 | ko04148 | Efferocytosis | 1 | 8 | 0.0556 | 0.022213 | 0.063911 |

Pathway enrichment analysis was performed using KEGG annotation. Matched metabolites (n) indicates the number of differential metabolites mapped to each pathway. Pathway size represents the total number of annotated metabolites included in the corresponding KEGG pathway. Rich factor was calculated as the ratio of matched differential metabolites to the total number of annotated metabolites in each pathway. P values were calculated using pathway enrichment analysis and adjusted using the Benjamini–Hochberg method.

**Supplementary Table 3. Ten-fold cross-validation performance of the Random Forest model with different mtry parameters.**

| **mtry** | **Accuracy** | **Accuracy SD** | **Kappa** | **Kappa SD** |
| --- | --- | --- | --- | --- |
| 2 | 0.930 | 0.091 | 0.862 | 0.179 |
| 145 | 1.000 | 0.000 | 1.000 | 0.000 |
| 289 | 1.000 | 0.000 | 1.000 | 0.000 |

Ten-fold cross-validation was performed using the caret framework in R. Accuracy represents the mean classification accuracy across 10 cross-validation folds, and Accuracy SD represents the corresponding standard deviation. The mtry parameter indicates the number of variables randomly sampled as candidates at each split during Random Forest model construction.

**Supplementary Table 4. Random Forest variable importance ranking based on mean decrease accuracy**

| **Rank** | **Metabolite** | **Importance score** |
| --- | --- | --- |
| 1 | Octadecanamide | 0.1086 |
| 2 | Oleamide | 0.0970 |
| 3 | 25-Hydroxycholesterol | 0.0916 |
| 4 | Artocarpin | 0.0345 |
| 5 | 2-Hexenoylcarnitine | 0.0247 |
| 6 | Indoleacrylic acid | 0.0218 |
| 7 | Lucidenic acid E2 | 0.0156 |
| 8 | 4-Vinylphenol sulfate | 0.0123 |
| 9 | alpha-Tocotrienol | 0.0106 |
| 10 | Tridemorph | 0.0103 |

Metabolites were ranked according to mean decrease accuracy derived from the Random Forest model with 1,000 trees. Higher importance scores indicate greater contribution to discrimination between CAVS patients and controls. This analysis was performed for exploratory feature prioritization.
